# A clinical algorithm to determine target blood pressure in the elderly: evidence and limitations from a clinical perspective

**DOI:** 10.1186/s40885-022-00202-9

**Published:** 2022-06-15

**Authors:** Jinho Shin, Kwang-il Kim

**Affiliations:** 1 Division of Cardiology, Department of Internal Medicine, Hanyang University Seoul Hospital, Hanyang University College of Medicine, Seoul, Republic of Korea; 2 Department of Internal Medicine, Seoul National University College of Medicine, Seoul, Republic of Korea; 3 Department of Internal Medicine, Geriatric Center, Seoul National University Bundang Hospital, 82 Gumi-ro, Bundang-gu, 13620 Seongnam, Korea

**Keywords:** Hypertension, Aged, Antihypertensives agents, Algorithms, Frailty, Hypotension

## Abstract

As the elderly population is growing rapidly, management of hypertension in South Korea faces major challenges because the proportion of elderly hypertension patients is also increasing. The characteristics of this population are also much more complex than younger patients. Elderly hypertension is characterized by wide variations in (1) fitness or biological age, (2) white-coat effect, (3) poor functional status or frailty, (4) dependency in activities of daily living or institutionalization, (5) orthostatic hypotension, and (6) multiple comorbidities. All of these should be considered when choosing optimal target blood pressure in individual patients. Recent randomized clinical trials have shown that the benefits of intensive blood pressure control for elderly patients is greater than previously thought. For generalization of these results and implementation of the guidelines based on these studies, defining the clinician’s role for individualization is critically important. For individualized decisions for target blood pressure (BP) in the elderly with hypertension, four components should first be checked. These consist of (1) the minimum requirement of functional status and capability of activities of daily living, (2) lack of harmful evidence by the target BP, (3) absence of white-coat hypertension, and (4) standing systolic BP ≥ 110 mmHg without orthostatic symptoms. Risk of decreased organ perfusion by arterial stenosis should be screened before starting intensive BP control. When the target BP differs among comorbidities, the lowest target BP should be given preference. After starting intensive BP lowering therapy, tolerability should be monitored, and the titration should be based on the mean level of blood pressure by office supplemented by out-of-office BPs. Applications of the clinical algorithms will be useful to achieve more standardized and simplified applications of target BP in the elderly.

## Background

Hypertension (HTN) is the most important controllable risk factor for cardiovascular (CV) events and mortality in the world [1]. There has been such progress in pharmacologic therapy that blood pressure (BP) can be controlled intensively under a target level below 120/80 mmHg, even in the setting of randomized clinical trials [2].

Aging is a global issue, and HTN is the second most common chronic disease or disability other than mobility impairment [3]. South Korea is the most steeply aging country, becoming an aged society in 2017. The elderly population aged ≥65 years comprises 16.7% of the national population. This proportion is increasing by 1% per year, resulting in a super-aged society within 10 years [4]. Among the 11 million hypertensive patients, 37.4% were elderly HTN patients in 2016. With a target BP of 140/90 mmHg, adherence is better than young patients, and the control rate of HTN in the elderly is about 60% [5]. Even though the elderly population is the largest target for HTN control, the threshold BP to be treated and the target BP achieved are still variable according to international guidelines. Moreover, the different target BPs according to comorbidity are frequently coexist in an elderly HTN patients [6–9]. The degree of frailty makes it difficult to choose the best threshold or target BP in an individual patient [10]. This review will discuss the components necessary to choose optimal target BP and the integration of these components to generate a useful clinical algorithm for elderly HTN patients with multiple comorbidities and/or frailty.

## Clinical problems of elderly hypertension

In a meta-analysis, elderly HTN is characterized by much higher absolute risk for CV events and mortality [11]. Despite the greater therapeutic benefit of BP reduction theoretically, the clinical benefit of BP control in elderly HTN was underrepresented by conservative approaches when the recent guidelines highlighting intensive BP control were introduced. With the need for more active BP control becoming accepted, clinicians have faced practical problems in implementing the target BP in the guidelines.

### Arterial stiffness and blood pressure variability

In the elderly, (isolated) systolic HTN is common, and it is closely related to increased aortic stiffness [12]. Aortic stiffness and vascular aging in elderly HTN are the key mechanisms of increased BP variability.

### White-coat hypertension

The prevalence of white-coat HTN (WCH) has been reported as 22.7% in a study of elderly HTN patients in clinics [13]. White-coat uncontrolled HTN was reported as reaching 30% among patients with uncontrolled clinical BP [14]. The prognosis of WCH and white-coat uncontrolled HTN are controversial but largely comparable to normal, and guidelines do not recommend pharmacological treatment [15]. Therefore, these two entities need to be excluded before starting or intensifying pharmacological treatment by using out-of-office BP measurement. If out-of-office BP measurement is not available, automated office BP (AOBP) can be measured as an alternative to diagnose WCH.

### Orthostatic hypotension

In the elderly, the prevalence of orthostatic hypotension (OH) is high (10–22%) and is associated with frailty, CV prognosis, and longitudinal cognitive function deterioration [16–18]. Dizziness as the representative symptom was reported as 40.6% in an in-hospital cohort [19]. However, OH was lower in patients with controlled BP [20], and intensive BP control was reported to be effective for longer survival in some studies, even in OH patients when it was tolerable [18].

### Concerns related to elderly hypertension

In addition to the white-coat effect (WCE) and OH, special considerations are needed for applying guidelines in the elderly, such as multiple comorbidities, frailty, and dementia or cognitive impairment [21].

### Gap between chronological versus biological age

There were reports regarding the indicators of biological age such as telomere length, epigenetic clock [22]. However, in a real practice, it is not uncommon to observe large individual variations in the gap between chronological and biological age. The critically associated factors or objective parameters to represent the gap are unknown. However, the findings that biological age or gap between self-perceived biological versus chronological age gap may be more predictive than chronological age itself, suggests that individualized assessment may be beneficial for BP target determination [23, 24].

### Recent target diseases for intensive blood pressure lowering therapy

Recent HTN clinical trials for target BP more frequently include the end points such as heart failure and dementia prevention [25, 26]. Recent heart failure guideline recommended the target BP to prevent heart failure with preserved ejection fraction as below 130/80 mmHg [27].

## Current target BP in guidelines for the elderly

### General target blood pressures in the elderly

There are large discrepancies among guidelines in terms of target BP in the elderly. In the 2017 American College of Cardiology/American Heart Association HTN guidelines, 130 mmHg of systolic BP (SBP) was recommended [6]; However, in the 2021 American Association of Family Physician guidelines, 150 mmHg was recommended as the general target for HTN in the elderly [7]. In the 2018 European Society of Cardiology/European Society of HTN [8] and 2018 Korean Society of HTN guidelines [9], 140 mmHg was recommended. In Canadian guidelines, an SBP target in AOBP < 120 mm Hg was proposed for all individuals aged over 75 years [28] (Table 1).

**Table 1:** Target blood pressure of older patients recommended by national guideline

Age (years)	KSH 2018	ACC/AHA 2017	ESC/ESH 2018	JSH 2019
≥ 65	140 (SBP) mmHg	130 (SBP) mmHg	130–139/70–79 mmHg	
≥ 75				140/90 mmHg

*KSH* Korean Society Hypertension, *ACC/AHA* American College of Cardiology/American Heart Association, *ESC/ESH* European Society of Cardiology/European Society of Hypertension, *JSH* Japanese Society of Hypertension, *SBP* systolic blood pressure

### Target blood pressure according to underlying comorbidities

In patients with a transient ischemic attack or stroke, the target BPs differ among the guidelines mainly because the benefits of the lower target are not supported by clinical trial evidence. However, more recent guidelines consider 130 mmHg, previously considered for lacunar infarction, as the general target BP [29]. Concerns for the potential harmful effect of intensive target BP were not validated in clinical trials such as Perindopril Protection Against Recurrent Stroke Study (PROGRESS) [30]. However, there was still an increased risk of adverse events in a recent observational study for the elderly [31]. Considering physiological mechanisms, the potential risk will be greatest in the patient having stroke history and multiple large artery stenosis.

In diabetes mellitus patients, 140 mmHg is suggested as the general target, but target BPs can vary according to the underlying risk profiles; for example, 130/80 mmHg was suggested as the target BP for high atherosclerotic CV disease risk, the presence of CV diseases or chronic kidney diseases [2, 32]. The rationale for the different target BP is not based on the risk of harm as shown in stroke history with multiple large artery stenosis but on the lack of clinical trial evidence for the benefits.

In chronic kidney diseases, the 2021 Kidney Disease: Improving Global Outcomes guidelines recommended < 120 mmHg in SBP as the general target BP if it is tolerable and standardized BP measurement is available considering Systolic Blood Pressure Intervention Trial (SPRINT) [33, 34]. The initial decline of the estimated glomerular filtration rate was not harmful for long-term renal prognosis. Therefore, how to define tolerability is clinically very relevant and important to decide target BP in an individual patient, and the factors related to the tolerability can be multiple and variable in a patient, as shown in Table 2.

**Table 2:** Factors related to tolerability in intensive blood pressure control

Factor	Clinical features
Functional status or physical fitness	Frailty, disability
Symptomatic aspects	Weakness, dizziness, fatigue
Diagnostic aspects	Standardized office blood pressure measurement Exclusion of orthostatic hypotension: orthostatic blood pressure measurement Assessment of white-coat effect or masked effect Application of home or ambulatory blood pressure monitoring
Hemodynamic aspects	Orthostatic hypotension Volume depletion or poor oral intake Presence of vascular stenosis in coronary, renal, and/or cerebral arteries
Related clinical event history	Injurious fall Acute kidney injury Electrolytes abnormalities
Speed of up-titration	Large pulse pressure Frail patients

## Tolerability defined in elderly hypertension trials

In the HTN in the very elderly trial (HYVET), patients aged > 80 years with a sitting SBP ≥160 mmHg were enrolled [35]. Patients with a standing SBP of less than 140 mmHg were excluded. In other words, OH, regardless of the presence of symptoms, was excluded. However, there was a substantial WCE at baseline and follow-up of 40 and 20 mmHg, respectively. Achieved daytime SBP was 126 mmHg [36]. But in the SPRINT [37], OH was also excluded only when SBP was < 110 mmHg, and greater than 95% of eligible OH were asymptomatic [38]. In addition, WCH was excluded by using AOBP [37]. Achieved daytime SBP was the same as HYVET, 126 mmHg [39]. In the Strategy of Blood Pressure Intervention in the Elderly Hypertensive Patients (STEP) trial, the achieved home BP in the intensive BP lowering group was close to 130 mmHg [26].

However, in an observational study for community-dwelling elderly HTN patients with 120 / 80 mmHg as the target BP, a 5-fold increase in injurious falls and syncope was reported compared to SPRINT [40]. This difference was explained by more frequent OH and comorbid CV diseases. However, in SPRINT, frailty, in which OH and WCH were excluded, showed no difference in the observed outcome in the elderly ≥75 years according to the grade of frailty [41]. Further analysis for side effects of the intensive BP control in SPRINT showed that the time to benefit was 1 year, and the time for harm was 3 months [42]. However, the mechanism of heterogeneity on the individual level regarding who will benefit or be harmed is unknown [43]. Therefore, to apply intensive target BPs by randomized controlled trial (RCT) evidence to real practice safely, exclusion of symptomatic OH and WCH, and initial close monitoring for side effects are essential.

## Components of the clinical algorithm for choosing the target blood pressure

### Four elements for the tolerability

#### Functional capacity

Frailty is a multidimensional syndrome of physical, cognitive, physiological, and social function decline whereas functional status in the conventional definition is restricted to physical function. There are many challenges to comprehensively define frailty. The clinical frailty scale, which was developed from the Canadian Study of Health and Ageing, can easily represent the frailty status of older patients [44]. Even though functional status and autonomy will drastically decrease in the 80s, in many patients, functional status and autonomy will still be preserved so they are relatively younger in terms of biological age. Physicians care for patients with a large spectrum of functional status and autonomy compared to those involved in RCTs, which show the typical benefits of intensive BP lowering. Therefore, some criteria to filter patients who do not benefit from antihypertensive medication (AHM) therapy or intensification of AHM are required. It is desirable to avoid aggressive therapies targeting only life prolongation in patients who lack independence, are bedridden, and/or institutionalized. In addition, deprescribing may be needed for residents in nursing homes [45].

To more effectively care for this population, the minimal requirement for starting AHM or escalating AHM needs to be defined. Benetos et al. [46, 47] suggested the threshold be “people living in nursing homes or needing assistance for activities of daily living (ADL)” because they were excluded from RCT and frequently showed a negative correlation between BP levels and life expectancy.

Preserved functional status with routine walking is essential to start intensive BP control. Residence in nursing home was an exclusion criterion, but wheelchair dependency itself was not an exclusion criterion for SPRINT. Slowing in routine walking itself cannot exclude the benefit of intensive BP lowering [48]. However, even with preserved autonomy for ADL, patients with a loss of functional status were excluded from HYVET. Chronic dependency on a step or wheelchair with preserved autonomy for ADL is a grey zone. It is considered that SPRINT can be applied to 64% of US population aged ≥ 75 years [49]. This report suggests that there is a sizable population that is not to be considered for intensive BP control in the population ≥ 75 years.

Until now, frailty status has not been considered when we start or intensify AHM. However, considering the importance of frailty status in managing older hypertensive patients, a frailty assessment should be included for the initial evaluation. Simple tests evaluating frailty status, such as gait speed, questionnaire in Korea, the so-called K-FRAIL (Korean version of the fatigue, resistance, ambulation, illnesses, and loss of weight) score, and the clinical frailty scale, can be used as screening tools for evaluating the frailty status of older patients [44, 50, 51].

#### Lack of evidence for benefits versus evidence of harm

Even though all medications carry an intrinsic risk of side effects and drug interactions, especially in older patients, most evidence of harmful effects used in RCT have been serious ones, such as major adverse CV events or functional deterioration of the organ levels. According to the “First do no harm” principle, clinical trial evidence of serious harm is a contraindication for intensive target BP, which has proved to be beneficial in the other comorbidities. Evidence of harm can be exemplified as a J-curve phenomenon in multi-vessel coronary artery stenosis or combination therapy using both an angiotensin converting enzyme inhibitor and angiotensin receptor blocker.

Without evidence of such serious harm, the higher target BP recommended just by the lack of benefit in one comorbidity can be disregarded when benefits of the lower target BP in other comorbidities were proved by RCT, as shown in Table 2. However, with evidence of harm, the target BP causing obvious harm in one comorbidity provides a contraindication for the more intensive target BPs in the other comorbidities.

**Fig. 1:**
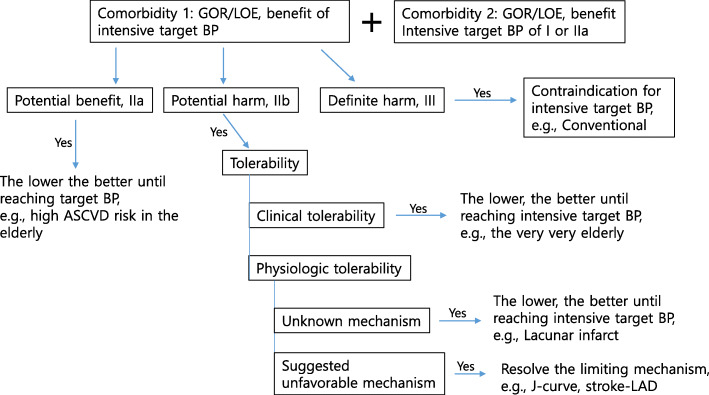
Individualized algorithm for target blood pressure (BP) according to different tolerability profiles in patients with multiple comorbidities. ASCVD, atherosclerotic cardiovascular diseases; CKD, chronic kidney diseases; e.g., for example; LAD, large artery disease. ^a)^Grade of recommendation: IIa, should be considered in favor of usefulness/efficacy; ^b)^IIb, may be considered with less-well established efficacy; ^c)^III, not recommended

In terms of the evidence of harm, most of the current evidence for serious harm is presumptive but inconclusive. As shown in Fig. 1, for a clinical situation facing this potential harm, tolerability to an intensive target could be categorized into greater physiologic tolerance to a specific organ or into a general clinical tolerance. For physiologic tolerance, it could be further categorized into those with a rather clear unfavorable mechanism or those with an obscure or unknown mechanism. For patients with general clinical tolerability issues or physiologic issues with obscure or unknown mechanisms, a lower target BP, which has proven to be beneficial by the presence of specific comorbidity, could be chosen. For this type of patient, titration could be carried out under close monitoring and a slower titration schedule. However, for patients with a physiologic tolerance issue with a clear mechanism, the underlying mechanism could be resolved before starting the intensive BP control. For example, in patients with diastolic BP < 70 mmHg concerned with a J-curve phenomenon, an evaluation for coronary artery stenosis followed by a revascularization therapy might be desirable [52, 53]. As another example, for a stroke patient with a large artery stenosis, a specialist consultation can be considered for the need of revascularization before starting intensive BP lowering or a patient-specific tolerable target BP. [54].

#### White-coat hypertension or white-coat effects

Unless WCH and/or WCE are excluded by AOBP, home BP monitoring (HBPM), or ambulatory BP monitoring (ABPM), BP control to the intensive target cannot be safely implemented for elderly HTN patients. If WCH and/or WCE cannot be excluded, the SBP target should be conservative and < 150 mmHg as long as the standing SBP is ≥140 mmHg at the time of starting AHM, as shown in HYVET in patient aged over 80 [35].

#### Orthostatic hypotension and the presence of symptoms

At the time of starting AHM, only patients with an asymptomatic standing SBP ≥110 mmHg should receive intensive target BP with the exclusion of potential WCE, as shown in SPRINT [35, 38]. Otherwise, standing SBP should be ≥140 mmHg at the time to start AHM therapy for the elderly > 80 years as shown in HYVET. In this case, the target SBP is 150 mmHg [35].

Therefore, routine screening of OH by measuring standing BP and by its symptom is critical. Typical orthostatic symptoms include dizziness, lightheadedness, blurred vision, weakness, nausea, and palpitations within a few minutes of standing [55]. OH can be transient during postprandial or it can be aggravated by dehydration, smoking, chronic kidney diseases, or medications [48]. Some patients with a standing clinical SBP > 110 mmHg in a morning session can experience postprandial orthostatic symptoms in the afternoon. In SPRINT, OH was associated with hypotension-related visits and bradycardia, but these associations were found not to be different between treatment groups [38]. OH was not associated with syncope, electrolyte abnormalities, injurious falls, or acute renal failure [38]. As long as orthostatic SBP ≥ 110 mmHg and asymptomatic, intensive BP lowering will be beneficial and asymptomatic OH during HTN treatment should not be regarded as a reason for down-titration even in the setting of intensive BP lowering [38]. Despite using more AHMs including chlorthalidone, intensive treatment of HTN lowered risk of OH, which is 20-fold more frequent in intensive treatment group than standard group. This is the case even though nonmyocardial infarction ACS was 2.5 times more frequently observed in the intensive BP lowering group [37, 38, 48]. Therefore, intensive BP lowering may not be applied for OH patients with (1) orthostatic symptoms, (2) a standing SBP of < 110 mm Hg, (3) diabetes mellitus, (4) prior stroke, or (5) dementia, representing the most severe cases of OH.

### Competing target blood pressures among comorbidities

**Fig. 2:**
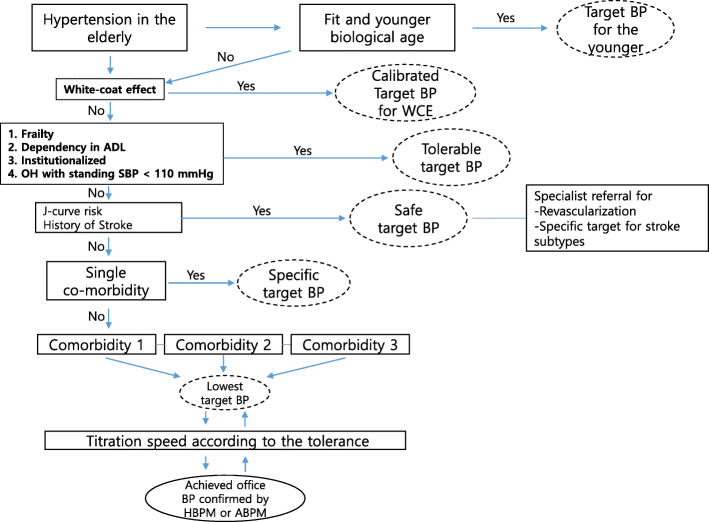
Practical algorithm for target blood pressure in individual elderly patients according to tolerability factors and the different target BPs among multiple comorbidities. ABPM, ambulatory blood pressure monitoring; ADL, activities of daily living; BP, blood pressure; HBPM, home blood pressure monitoring; OH, orthostatic hypotension; SBP, systolic BP; WCE, white-coat effect

If there is no evidence of harm, the lowest target BP should be chosen with the above mentioned four essential components screened and integrated for decision making, as shown in Fig. 2. Target BPs for the more recently introduced end points such as cognitive function and heart failure could be understood differently from the patient perspective. For example, the overall survival benefit could justify intensive BP lowering as long as the impact on individual organ functions was neutral or uncertain in some patients [37, 48]. Therefore, intensive BP control definitely associated with deterioration of some organ functions that are unacceptable for a patient will not be adopted, even though intensive BP control could improve survival itself and vice versa (Table 3).

**Table 3:** Algorithm to choose target blood pressure (BP) to improve global outcome according to the grade of recommendations for specific target BP in the elderly patient with three comorbidities with different grades of recommendation for target BP

Case	Comorbidity 1	Comorbidity 2	Comorbidity 3	Target BP
1	I	I	I	Lowest target BP among the comorbidities 1, 2, and 3
2	IIb	I	I	Lower target BP between the comorbidities 2 and 3
3	IIb	IIa	I	Target BP according to the comorbidity 3 or the lower target BP between the comorbidities 2 and 3
4	III	I	I	Target BP according to the comorbidity 1

I, recommended; IIa, should be considered in favor of usefulness/efficacy; IIb, may be considered with less well-established efficacy; III, not recommended

### Monitoring and titration

During intensive BP control for potential benefit without RCT evidence, for example, intensive BP lowering in stroke patients only showing benefit in SPRINT eligible lacunar infarct patients in post-hoc analysis of the SPS3 trial, close monitoring for side effect is mandatory [56]. When intensive BP lowering is inevitable because of comorbidities such as symptomatic heart failure, close monitoring organ functions such as acute kidney injury or neurologic signs is essential.

The speed of titration of AHM to achieve the target SBP can vary among patients to patients. All side effects including symptomatic OH and the impact of intensive treatment on functional status and ADL should be routinely checked during follow-up. In elderly patients > 80 years, reduction of weight, oral intake, physical activity, or cognitive function should be closely assessed because sarcopenia, dental problems, mental health problems, and cachexia can be common during the natural aging process. Programmed physical training and a supply of adequate protein and calories should be considered positively. Whether excessive reduction of salt intake could result in a reduction of oral intake and/or weight should also be assessed. During the monitoring period, in patients in whom intake and weight are decreasing, de-escalation of AHM can be based on the level of standardized office BP. The presence of symptomatic OH with uncontrolled office BP does not necessarily mandate immediate down-titration for older, community-dwelling hypertensive patients and out-of-office BP measurement will be useful for titration.

## Korean perspectives

### Atherosclerotic cardiovascular disease risk driven target blood pressure

In SPRINT, patients with a 10-year risk of CV disease of 15% or greater on the basis of the Framingham risk score were enrolled. For Korean patients, the risk stratification equation is reported to be much more dependent on age so that most of the hypertensive elderly are classified as a high-risk group. If there are no limiting factors for tolerability, there is greater opportunity to try intensive BP control in Korean elderly patients. However, because of the limitations of the current risk stratification system for Koreans, more evidence for an atherosclerotic CV disease risk-based strategy for intensive BP lowering is needed. In addition, the risk stratification model overestimates the CV risk of Asian patients [57]. Accordingly, applying the Framingham risk score or other risk models results in overtreatment for older Asian patients [58]. A new risk stratification model for Asian people should be developed to accurately assess the risk of hypertensive patients.

### Accurate blood pressure measurement

Exclusion of the WCE or WCH is mandatory to make the decision for intensive BP lowering in the elderly. In general, strictly standardized office BP measurement is sufficient for starting and monitoring intensive BP lowering. In the case of the elderly, however, it is reasonable to use HBPM or ABPM to ensure the safety of vulnerable patients. When HBPM or ABPM is not available, AOBP can be useful to exclude WCH at the time of diagnosis of HTN. In Korea, routine AOBP does not seem to be feasible because of limited space in the clinic. Practical solutions for HBPM or application of smart devices in clinical practice are useful to determine individualized target BP in the elderly.

Once a patient for intensive BP control is selected by clear screening of the four elements of tolerability, clinicians need to know that both the research level office BP measured using automated device and AOBP had a significant masked effect greater than 5 mmHg when SBP was maintained at around 120 mmHg safely, as shown in the SPRINT and STEP study. When BP is maintained at 130 to 135 mmHg, HBPM and daytime ABPM are the most equivalent to office BP or AOBP [59].

### Hypertension management for frail or institutionalized patients

With population aging, we should prepare for the increase of the oldest old or frail elderly patients. Frail older adults are highly vulnerable to external stresses. Thus, they are high-risk groups for adverse CV events. Frailty is common among institutionalized older adults. Previously, it has been reported that the prevalence of frailty and pre-frailty in nursing homes was 52.3% and 40.2%, respectively [60]. It remains unclear what optimal treatment for hypertensive patients with frailty would be. In particular, an optimal management plan for institutionalized patients has not been published. There were concerns that too low BPs, which were common among frail or institutionalized patients, were associated with excess mortality or increased CV risk. Accordingly, current guidelines recommend more conservative antihypertensive treatment (i.e., fewer AHMs) for frail or institutionalized older patients. However, a previous study showed that BP tended to decrease in the final two years of life, which suggests a reversal of causality between low BP and adverse clinical outcomes [61]. Therefore, further studies are required on whether deprescribing or withdrawal of antihypertensive drugs has any beneficial effect on the clinical outcomes of older hypertensive patients.

## Conclusions

In conclusion, for individualized decisions for target BP in the elderly with hypertension, using a clinical algorithm considering functional status, WCH, OH, evidence of harm and multiple comorbidities will be useful to achieve more standardized and simplified applications of target BP in the elderly.

## Data Availability

Not applicable.

## Abbreviations

ABPM: Ambulatory blood pressure monitoring
ADL: Activities of daily living
AHM: Antihypertensive medication
AOBP: Automated office blood pressure
BP: Blood pressure
CV: Cardiovascular
HBPM: Home BP monitoring
HTN: Hypertension
HYVET: HTN in the very elderly trial
KSH: Korean Society Hypertension
OH: Orthostatic hypotension
RCT: Randomized controlled trial
SPRINT: Systolic Blood Pressure Intervention Trial
WCE: White-coat effect
